# Supplementary material for: Chromothripsis during telomere crisis is independent of NHEJ, and consistent with a replicative origin
Source: Genome Res. 2019 May;29(5):737–49. doi: 10.1101/gr.240705.118 (PMC6499312; doi:10.1101/gr.240705.118)
Supplement: Supplemental Material [file supp_gr.240705.118_Supplemental_file_1.zip › contigs/annotated_contigs/DB46/contig.2.DB46_length_453_mean_cov_3.86975717439.docx]

**DB46_length_453_mean_cov_3.86975717439**

```
AGACACAGAATATATATGTGTATATTTATGTATATATATGTGTGTGTGTATATGTGTGTGTGTTTATATTTATATATATATATAAACCT
 >chr14:86785200-86785567 + E=2e-202
AAACTGCATCATAGCATTTGACAGTGTAATTTGAAAGAAAAAAAAAAACTCTGTATTTCCGTGATAGTGAAATATCTAAATTAACTATA

AGATGGTAATGCTATATGATGTTCCATATGTCAATTATAAATAGACAAATTGGTTTTAGTTATATATTAAGGGAAGTATTTTATGCATA

TGCTTCAAAACTATTTACATAAAAAGTTACCGAATTGTATTATTTTATAAGGATACATATATATATATGTATATATATATATATATATA

TACTATATATAT|ATA|CTTTCGCATGCTATTCTAATGATATGGAATAAGTCTCAAAAGATCTGATGACGTCATAAAGGGGAGATTCAC
                >chr14:86786883-86786965 - E=1e-16
TGCACAAATT
```
